# Perspectives of Post-Acute Transition of Care for Cardiac Surgery Patients

**DOI:** 10.3389/fcvm.2017.00070

**Published:** 2017-11-27

**Authors:** Nicoleta Stoicea, Tian You, Andrew Eiterman, Clifton Hartwell, Victor Davila, Stephen Marjoribanks, Cristina Florescu, Sergio Daniel Bergese, Barbara Rogers

**Affiliations:** ^1^Department of Anesthesiology, The Ohio State University Wexner Medical Center, Columbus, OH, United States; ^2^The Ohio State University College of Medicine, Columbus, OH, United States; ^3^University of Medicine and Pharmacy Craiova, Craiova, Romania; ^4^Department of Neurological Surgery, The Ohio State University Wexner Medical Center, Columbus, OH, United States

**Keywords:** transition of care, cardiac surgery, post-acute care, patient outcome, skilled nursing facilities, hospice

## Abstract

Post-acute care (PAC) facilities improve patient recovery, as measured by activities of daily living, rehabilitation, hospital readmission, and survival rates. Seamless transitions between discharge and PAC settings continue to be challenges that hamper patient outcomes, specifically problems with effective communication and coordination between hospitals and PAC facilities at patient discharge, patient adherence and access to cardiac rehabilitation (CR) services, caregiver burden, and the financial impact of care. The objective of this review is to examine existing models of cardiac transitional care, identify major challenges and social factors that affect PAC, and analyze the impact of current transitional care efforts and strategies implemented to improve health outcomes in this patient population. We intend to discuss successful methods to address the following aspects: hospital-PAC linkages, improved discharge planning, caregiver burden, and CR access and utilization through patient-centered programs. Regular home visits by healthcare providers result in decreased hospital readmission rates for patients utilizing home healthcare while improved hospital-PAC linkages reduced hospital readmissions by 25%. We conclude that widespread adoption of improvements in transitional care will play a key role in patient recovery and decrease hospital readmission, morbidity, and mortality.

## Introduction

The rapid growth of the geriatric population will trigger a necessary increase in utilization of healthcare resources, including the use of surgical specialties such as cardiac surgery; therefore, transition of care following cardiac surgery will continue to increase in importance in the coming years (1). Transition of care is defined by The American Geriatrics Society as the “set of actions designed to ensure the coordination and continuity of healthcare as patients transfer between different locations or different levels of care within the same location…. It includes the logistical arrangements, education of the patient and family, and coordination among the health professionals involved in the transition” (2). Per US Census Bureau, the older population will double between 2010 and 2050, with individuals older than 85 years representing the fastest growing group (3). The post-acute care (PAC) options covered by this review include skilled nursing facilities (SNFs), long-term acute care hospitals (LTACHs), cardiac rehabilitation (CR) centers, and the patient’s home.

Shared electronic health records (EHRs) facilitate patient transitions within the hospital. Recent reports indicate that an increased number of patients utilize PAC facilities at discharge; for example, 22.3% of discharged patients used PAC services in 2013, as an increasing trend since 2008 (4, 5). Since hospitals and PAC facilities are often run independently, utilizing different systems of operation and means to maintain patient records, effective communication and coordination remain a challenge, and could lead to “avoidable” treatment errors and readmissions (6).

There is little shared health record information between institutions, perhaps partially due to many PAC facilities not utilizing EHRs at the same rate as acute care settings, and the inability of both acute care and PACs to exchange clinical data with external institutions.

Many hospitals and PAC settings have not established a partnership where these challenges could be addressed more directly (6).

Strategies to improve transition of care such as hospital-PAC linkages, improved discharge planning, increased access and referrals to CR programs, and more effective use of electronic records are being tested but have not seen widespread adoption. Our review aims to explore the current impact of transitional care on outcomes for cardiac surgery patients, inadequacies in accessibility and affordability, and current efforts to address these issues.

## Cardiac Surgery Etiology and Demographics

Cardiovascular disease (CVD) is the number one cause of death (614,348 deaths) in the United States based on a census published by the CDC in 2014, with a prevalence up to 87.1% in the elderly population (7, 8). According to the US Census Report, approximately 14.5% of the population is 65 years of age and older and is projected to live an average of 19.3 years beyond 65 (~84.3 years old) (7). A significant percentage (20.3%) of this population will require a corresponding adjustment of healthcare assistance (9).

Cardiovascular disease and coronary heart disease can often progress to a point where cardiac surgery becomes the only treatment option. According to the National Hospital Discharge Survey, there were 3.891 million cardiac procedures in 2010 (10). In 2013 alone, 410,610 CABG, PCI, valve, and pacemaker heart surgeries were performed in patients older than 65, and this age group made up 13.0% of total hospital discharges (7). After discharge, patients are often referred to a PAC facility (Table 1) (11, 12). To ensure the best outcomes for this population, it is critical to understand factors that affect patient recovery after surgery, specifically the PAC transition.

**Table 1:** Current post-acute care options (11, 12).

Skilled nursing facilities (SNFs)	A transitional facility between hospital and home where patients receive intensive, short-term nursing care during recovery. SNF services may include wound care, administration of medication, dietary counseling, physical, occupational, and speech therapy
Home healthcare	A short-term, home treatment plan that includes a range of services such as wound care, physical and occupational therapy, medical social work, as well as regular visits from physicians, nurse practitioners, or nurses throughout the patient recovery
Long-term acute care hospital	A facility that cares for patients with more complex health issues requiring ≥25 days stay. Services include pain management, dialysis, occupational, speech and physical therapy, respiratory therapy, and head trauma treatment
Nursing home	A facility that focuses on providing custodial care for their residents, including basic activities for daily living
Cardiac rehabilitation	A program that incorporates patient assessment, exercise training, nutritional counseling, management of health issues, psychosocial support, and patient education. Services offered in inpatient, outpatient (nursing home/extended care facilities), and home-based settings
Hospice/palliative care	An end-of-life service that is focused on providing pain management, as well as physical, emotional, spiritual care, with the goal of making the patient’s life as comfortable and dignified as possible until his or her passing. This type of service is offered in hospice centers, hospitals, long-term care facilities, or at home

## PROS and CONS of PAC Settings

Dedicated PAC institutions provide specialized care based on the type and severity of the illness (Table 2 includes data from the four most relevant care settings) (12, 13). Utilization of PAC varies depending on region, hospital location and size, procedure type, and payer plan (Table 3) (4).

**Table 2:** Admission rates and illness severity corresponding to acute hospital care and different post-acute care settings (12, 13).

		Acute care hospital (%)	SNF (%)	HHC (%)	LTACH (%)
Severity (18)	Least severe	16.9	8.6	21.3	2.4
	Moderate	42.4	37.1	46.7	16.4
	Severe	31.8	41.8	28.0	38.0
	Most severe	9.0	12.5	4.0	43.3
Admission rate following cardiac surgery (19)		N/A	7.5	84.4^a^	4.4

*SNF, skilled nursing facility; HHC, home healthcare; LTACH, long-term acute care hospital*.

*^a^This value represents patients discharged home following cardiac surgery*.

**Table 3:** Determinants of post-acute care (PAC) facility usage (4).

More likely to use PAC facilities	Less likely to use PAC facilities
Urban teaching hospitals	Non-teaching urban or rural
Large hospitals	Medium or small hospitals
Southern hospitals	Northeast and Midwest hospitals. West has least utilization
Medicare is the primary payer for PACs (64.6–84.9% depending on the type of PAC)	Patients with private insurance or Medicaid, and those uninsured

### Skilled Nursing Facilities

Among Medicare beneficiaries in 2003, 11% of CABG patients and 20% of valve surgery patients received SNF care (14). In 2014, the Medicare spending on SNFs was $28.6 billion dollars, with 15,000 SNFs caring for almost 1.7 million Medicare beneficiaries (15). Medicare fully covers eligible patients for the first 20 days, and partially covers up to 100 total days in an SNF after a necessary inpatient stay (15). Medicaid can also help pay for long-term nursing home stays if the patient qualifies (16).

The vast majority of cardiac surgery patients are discharged home with the second most common destination being SNFs (Table 2) (13). In 2014, SNFs were able to improve patients’ activities of daily living (ADLs) by 43.5%, as well as rehabilitate and transition them home ranging from 37.3 to 47.3% depending on the SNF efficiency. Hospital readmission rates ranged from 10.9 to 9.2% for the same year, an improvement from recent years. Despite recent improvements (2011–2014)—when avoidable readmissions decreased by 12.1% and community discharge improved by 13.6%—a high rate of unplanned hospital readmissions and mortality continues to impact patient quality of care (15). Henry et al. (17). indicated that there was a 2.5 times greater mortality hazard, even after adjusting for age, gender, European System for Cardiac Operative Risk Evaluation, and complications, for octogenarian patients undergoing valve replacement surgery and discharged to an SNF versus home (18).

### Home Healthcare

Most patients undergoing cardiac surgery (84.4%) do not require further intensive nursing care and are discharged home (Table 2) (12). Another alternative for discharge is home healthcare (HHC). To qualify for HHC, the patient must be homebound and require intermittent nursing services. Surgical patients undergoing interventions such as CABG and cardiac valve replacements are more likely to qualify for HHC than medical patients undergoing treatments for heart failure and myocardial infarction, who generally transition to SNFs (14). The 1-year survival rate for HHC recipients is much higher (95.4%) compared with SNF patients (52.3%). This difference may be attributed to the fact that SNFs generally receive patients in more severe conditions than HHC (Table 3).

Hall et al. reported 3.9% 30-day readmission rates for HHC CABG patients who were visited regularly by a cardiac surgery nurse practitioner, compared with 11.5% readmission rates for patients who received no in-home visits (18). A recent study showed a 41% reduction in 30-day readmission of cardiac surgery patients and decreased healthcare costs when home visits were covered by a physician assistant (19).

### Long-term Acute Care Hospital

Long-term acute care hospitals are intended for patients with complex medical problems requiring a stay of more than 25 days (12). A study published in 2013 by Edgerton et al. concluded that LTACH patients have the lowest survival rates when compared with other PAC facilities (13). Based on severity of illness corresponding to levels 3–4 (most severe), approximately 81% of LTACH patients, 54% of SNF patients, and 32% of HHC patients meet the aforementioned criteria (Table 2) (12). Patients utilizing this service through Medicare generally are responsible for only one deductible per stay, with similar out-of-pocket costs as a typical acute care hospital visit (11).

### Nursing Home/Extended Care Facilities

Patients can also transition into nursing homes following cardiac surgery, but this can be a costly option. While Medicare generally covers care given in SNFs, LTACHs, and HHC, patients must pay for most nursing home services that are not medically necessary to rehabilitation. Sixty-three percent of patients receiving nursing home/extended care pay for it by personal sources alone, while 28% of care is paid through public programs (Medicare and Medicaid) and 9% through both public and personal funds (20). For patients with higher income or family financial support, this is not a major challenge and they are able to receive higher levels of care when compared with patients with lower income or less familial financial support (20).

### Cardiac Rehabilitation

Cardiac rehabilitation has been shown to be very effective in reducing hospital readmissions and improving ADLs for patients following cardiac surgeries (21). A physician must refer the patient to a program following a cardiac event or surgery. Medicare will cover up to 36 sessions over 12 weeks, with an additional 36 sessions if required (22). There are several types of CR, including inpatient, outpatient, and home-based.

Long-term results of CR are promising based on recent data. A study published in 2009 indicated that 1-year mortality rates were 43–58% lower for those patients who underwent CR (23). Patients that attended the most sessions tended to have the best outcomes; Shook et al. reported significantly lower 180-day readmission rates post-discharge for subjects participating in Phase II CR (23). A longitudinal 5-year data analysis indicated that 75% of patients with severe disability were able to improve functional status and was associated with a 50% decrease in mortality (24).

Despite its effectiveness, CR is underutilized by patients. A study published by Schuster et al. considered the lack of insurance coverage as the top reason for poor/non-adherence to the program (25). Even with Medicare, patients are responsible for up to 20% of copay, which can be a significant financial burden (26). Other studies have invoked additional contributory factors to underutilization: lack of physician referral or encouragement to participate, work/home responsibilities or conflict, scarcity of programs, low education about CR programs and cardiac disease, negative perceptions, or lack of familial support (27–29).

## Social Factors Associated with PAC

### Racial Disparities

Black patients and other minority patients are over-represented in poor-quality hospitals characterized by higher mortality rates (30). In addition, black patients are significantly more likely to be part of a lower socioeconomic group, present with more comorbidities, and be readmitted to the hospital following cardiac surgery (31). Compared with white patients, black patients were found to have similar 90-day mortality rates post-CABG, but higher 1-year mortality rates (32). This may be attributed to complications related to comorbidities or inadequate long-term care following surgery. In a study involving 23,000 CABG patients, Koch et al. reported similar results, with black patients and women having worse survival rates. However, short- and long-term survival rates were similar irrespective of race when controlling for comorbidities, perioperative factors, and socioeconomic status (SES) (33). Further study into the impact of race and strategies to combat this bias are required.

### Gender Disparities

Female patients undergoing CABG without concomitant valve surgery had additional comorbidities and more advanced age compared with males. Female gender was still a significant independent predictor of mortality (34). Women undergoing CABG were more likely to be discharged to an SNF, while men were 20–60% more likely to be discharged home (14, 35).

Women may have a more difficult time recovering from cardiac surgeries such as CABG, reporting higher symptom evaluation scores for fatigue, sleep disturbance, swelling, anxiety, and shortness of breath, along with lower physical function (35, 36). Barnason et al. also found that women had decreased ability to perform daily activities compared with men at 12 months after CABG (37).

According to a 1998 study, women used CR services significantly less than men (38). In recent years, women still are not receiving the cardiac rehab that they should. Lavie et al. estimate that only 5–10% of eligible patients attend at least one session of CR, and even fewer complete the entire program (39). They suggest that women face additional barriers when compared with men, such as fewer referrals to rehab and lower SES. Gender-related discrepancies in CR utilization could be addressed by providing women-only or women-tailored CR sessions, avoiding public weighing, and diversifying CR exercise options by offering yoga or dance (39). Schuster et al. consider that women may benefit greater from structured CR, as women at home showed decreased exercise adherence compared with men at home or compared with women in structured programs (27).

## Mental and Physical Health Associated with Post-Acute Cardiac Care

Older age is known to predict outcomes such as functional status, quality of life, and readmission to the hospital (14, 40, 41). Going beyond physical weakness, Neupane et al. advocate the use of the biopsychosocial framework to evaluate patients (42). They divide frailty into biological, psychological, and social domains.

Psychosocial factors are often overlooked. Cardiac surgery is frequently associated with mental stressors that affect patient outcomes (43). A systematic review published by Ravven et al. concluded that elderly patients undergoing CABG suffer from depression both preoperatively and postoperatively. After surgery, there was an early increase in the depression prevalence (35.5%), with an overall improvement in the next 6 months (21.6%) (44). Women are more susceptible to depression preoperatively than men (45). Thus, it is important to screen for depression both before and after surgery. Social factors that influence recovery included social support and SES. Married patients have been shown to have better outcomes than single or widowed patients (44). Other aspects such as religion and prayer may also be beneficial for patients (46, 47).

### Caregiver Financial Burden in Transition of Care

Given the high percentage (63%) of patients paying out of their own pocket, it would be reasonable to conclude that limited funds may lead to limited/insufficient care. In fact, the same study also demonstrated that older patients with family incomes greater than $75,000 receive 8.5 more hours of home care compared with patient families in the lowest income category, $15,000 (20). Patient families in the $75,000 income category are receiving more care, but they are also paying for more care out of pocket. About 97% of home care received by these patients was paid for by personal funds alone, as opposed to using any public programs. Patients in the lower income bracket who are more dependent on publicly funded programs may receive less professional care, and must rely on themselves or family members for assistance.

## Current Efforts in PAC

### Hospital-SNF Linkages

The degree of linkage is determined by the proportion of patients from the originating hospital who were discharged to the treating SNF (48, 49). Highly linked SNFs are usually closer in distance to the originating hospital, and there may be better communication and protocols when transferring patients or when issues arise. For example, at Cedars-Sinai Medical Center in Los Angeles, nurse practitioners coordinate with both hospital and SNF teams to follow-up on patient issues. Since implementation, they have noticed a 25% drop in the readmissions rates and a reduction of medication errors in SNF patients (50).

Hospitals can affiliate with better SNFs to ensure the best patient outcomes, while SNFs will be incentivized to improve the quality of care to maximize their hospital referral rates (15). According to Medicare data, quality among SNFs can vary greatly. In their 2014 analysis, only 8% (892 of the 11,637 facilities) were deemed to provide relatively low-cost, high-quality care by the Medicare standards, an increase from 7% in 2013 (15). When the SNFs were ranked by community discharge rate, the commission found that lowest quartile had community discharge rates of lower than 29%, while the best SNFs in the upper quartile had rates of 46% and higher. Other measures with the same pattern include readmission rates and ADLs rating. However, it is important to consider a holistic approach. After adjustment for patient factors, SNF facility factors, and discharging hospital, Neuman et al. concluded that SNF performance measures were not consistently associated with lower adjusted risk of readmission or death after 30 days (51).

Skilled nursing facility organizational structure influences quality of care through enhancing team coordination. The most effective SNF focused on the following strategies: (1) promoting staff knowledge so that each employee understood his or her role in transitioning patient; (2) providing instruction to patients regarding self-managed care at home; (3) routinely held meetings to coordinate and plan for patient needs at home; (4) using standardized templates when documenting in patient EHRs; and (5) maintaining a proactive, problem-solving approach by interdisciplinary teams. The poorest performing SNFs did not consistently assess patient needs at home, reconcile medication lists, engage primary caregivers, teach written transition plans, or provide detailed enough written instructions for home care (52, 53).

### Improved Discharge Planning

Altfeld et al. reaffirmed the importance of psychosocial factors in overall readmission rate (40–50%) (54). They designed an at-risk patient protocol influenced by previous models consisting of a phone-based assessment and planning for patients after home discharge. The study included patients that were living alone, lacking a support system, at risk for readmissions or falls, or with a high-psychosocial need based on staff assessment. The study concluded that 83.3% of patients had significant barriers to care, with 73.3% presenting with post-discharge barriers. The results emphasize that follow up care is critical as unexpected problems often arise after discharge (54). Of those patients, 45.8% experienced problems involving self-management of care. Other identified barriers include caregiver burden (35.0%), delivery of home health services (25.6%), obtaining community resources (23.6%), and coordination between providers (19.4%). The authors believed social workers may remedy some of these issues by providing emotional support, communication between patient and providers, and addressing needs beyond the scope and purpose of the acute hospital discharge planning process (54).

An anesthesiologist-led multidisciplinary, team-based care model known as the perioperative surgical home (PSH) provides surgical patients with stable continuity of care (55–57). The PSH aims to improve patient outcomes by standardizing best practices, increasing patient-centered continuity of care, and decreasing perioperative costs (55, 57–59). Integrated care plans have been shown to decrease length of stay, in-hospital complications, and reduce hospital costs (60). According to Jones et al., several studies have shown benefits of developing a comprehensive discharge checklist for post-surgical patients, leading to improved patients outcomes through reduced readmission rates. Project re-engineered discharge used a checklist to manage consults, follow-up appointments, discharge equipment, and medications after discharge, leading to decreased 30-day readmission rates (61).

Another strategy that could improve discharge planning is the utilization of an “e-health” platform. Cook et al. recently reported their experience administering electronic tablets to patients to provide feedback to the hospital staff regarding pain and discomfort, mobility progress, and early discharge screening. The authors point out that shorter lengths of stay in the hospital were correlated with earlier patient-reported mobility in the post-operative recovery process. In addition to this, they implemented “early screen for discharge planning” (ESDP), which generates an ESDP score; a higher score was significantly associated with longer stays in the hospital, as well as an increased likelihood of being discharged to an SNF or HHC. In their model, patients were also informed (*via* their iPads) of daily recovery expectations. The authors concluded that “direct patient self-assessment and reporting using mobile computing” will simplify the communication between the hospital and patient during recovery (62).

### Addressing Caregiver Burden

Hospital post-discharge strategies aim to assist patient care and alleviate caregiver burden through pre-discharge planning/education and telehealth care (63, 64). A study published by Ruiz et al. suggested that caregiver attitude may affect patient outcome. During an 18-month period, 111 male CABG patients and their spouses completed measures of neuroticism, optimism, perceived marital satisfaction, and depression (pre-/post-intervention). The authors concluded that higher *caregiver* pre-surgical neuroticism predicted higher *patient* depressive symptoms and higher *patient* pre-surgical neuroticism is associated with higher *caregiver* depression at follow up visit (65). Additional support for caregivers should focus on their new, challenging responsibilities, including tasks inside and outside the home, emotional support, transportation, and monitoring and reporting symptoms (66). Lastly, preoperative patient education is known to decrease patient-reported anxiety and depression and to improve subjective health and outcomes (67).

### Strategies to Improve CR Program Access

Finally, improving CR programs/access remains a crucial part of the patient recovery process. Hospital-level factors significantly influence CR referral rate with physician referral being one of the strongest predictors for program enrollment (28, 68). Automatic referral systems and staff liaisons were effective in the CR enrollment process, while a combination of both increased the likelihood of CR referral eightfold; however, a recent survey of CR program directors suggested underutilization of CR referral strategies (21, 69).

A randomized control pilot trial tested additional strategies to increase CR enrollment, such as peer navigation (PN) and an eReferral system. PN employs a trained layperson to work one-on-one with patients and provide education about the healthcare process and support. Nevertheless, PN did not increase enrollment rates. The eReferral system utilizes a computer prompt on cardiac patient discharge summaries asking the provider if the patient should be referred to CR. They considered that this process mitigated “sociodemographic biases oft-observed in CR referral.” Lastly, they concluded that higher rates of CR enrollment occurred when patients were referred to the CR sites closest to their homes (70).

Another issue that needs addressing is that CR programs are not equally distributed across the United States, with up to ninefold differences in utilization among states (71). The highest rates of enrollment are reported in the northern Midwest states and the lowest rates in the South, possibly corresponding to the population density and geographic distribution of CR programs (72). Telemedicine-based CR could provide a viable and affordable alternative for patients unable to access CR centers (73). Taylor et al. performed a secondary analysis of 17 trials in 10 countries on patients undergoing CR, concluding that home and center-based rehabilitations showed no significant difference after 1 year in terms of mortality, cardiac events, or exercise capacity (74). When utilized correctly, home-based and center-based CR can be equally effective.

## Conclusion

Seamless transition of care is becoming increasingly important for high-risk patients, undergoing major cardiovascular-related surgeries since the rates of CVD are rising in the United States’ aging population.

Transition of care is influenced by a myriad of factors, including severity of illness at discharge, the type of PAC used, efficient transition between hospital and PAC setting, adherence to PAC services, caregiver burden, patient psychosocial support, and SES. The use of telemedicine, automatic/physician referrals, team-led or structured models for discharge planning and hospital-PAC linkages, and patient follow up by a healthcare professional are implemented in order to improve patient outcome.

Patient-centered programs focused on transition of care and improved CR utilization should be further tested in order to minimize hospital and patient financial burden through reduced readmission, morbidity, and mortality.

## Author Contributions

All authors listed, have made a substantial, direct and intellectual contribution to the work, and approved it for publication.

## Conflict of Interest Statement

The authors declare that the research was conducted in the absence of any commercial or financial relationships that could be construed as a potential conflict of interest.
